# Chrysoxanthones A–C, Three New Xanthone–Chromanone Heterdimers from Sponge-Associated *Penicillium chrysogenum* HLS111 Treated with Histone Deacetylase Inhibitor

**DOI:** 10.3390/md16100357

**Published:** 2018-10-01

**Authors:** Xin Zhen, Ting Gong, Yan-Hua Wen, Dao-Jiang Yan, Jing-Jing Chen, Ping Zhu

**Affiliations:** State Key Laboratory of Bioactive Substance and Function of Natural Medicines, Key Laboratory of Biosynthesis of Natural Products of National Health Committee, Institute of Materia Medica, Chinese Academy of Medical Sciences and Peking Union Medical College, 1 Xian Nong Tan Street, Beijing 100050, China; zhenxin@imb.pumc.edu.cn (X.Z.); gongting@imm.ac.cn (T.G.); wenyanhua@imm.ac.cn (Y.-H.W.); yandaojiang@imm.ac.cn (D.-J.Y.); chenjingjing@imm.ac.cn (J.-J.C.)

**Keywords:** *Penicillium chrysogenum*, secondary metabolites, histone-deacetylase inhibitor, antibacterial activity, polyketide synthase

## Abstract

By treating with histone-deacetylase inhibitor valproate sodium, three new heterdimeric tetrahydroxanthone–chromanone lactones chrysoxanthones A–C (**1**–**3**), along with 17 known compounds were isolated from a sponge-associated *Penicillium chrysogenum* HLS111. The planar structures of chrysoxanthones A–C were elucidated by means of spectroscopic analyses, including MS, 1D, and 2D NMR. Their absolute configurations were established by electronic circular dichroism (ECD) calculations. Chrysoxanthones A–C exhibited moderate antibacterial activities against *Bacillus subtilis* with minimum inhibitory concentration (MIC) values of 5–10 μg/mL.

## 1. Introduction

The *Penicillium chrysogenum* species is commonly used in the industrial production of *β*-lactam antibiotics. This species is present in various habitats including plants, gorgonians, marine sediments, algae, sponges, and mangroves. The whole genome of the strain *P. chrysogenum* Wisconsin 54-1255 was sequenced in 2008, and up to 33 secondary metabolic gene clusters were predicted, showing huge potential to produce various types of compounds in addition to *β*-lactam antibiotics [1]. However, limited products with six chemical types were obtained from *P. chrysogenum*, much fewer than the estimated number that the secondary metabolic gene clusters could generate. Therefore, it is believed that many of the secondary metabolic gene clusters may be silent in laboratory culture conditions. In recent years, strategies related to epigenetic modifications, such as the addition of a histone-deacetylase inhibitor and disruption of gene-encoding histone deacetylase (HDAC) have been proven to be able to effectively activate the silent biosynthetic gene clusters and lead to the formation of diverse secondary metabolites in fungi [2,3,4,5].

**Figure 1:**
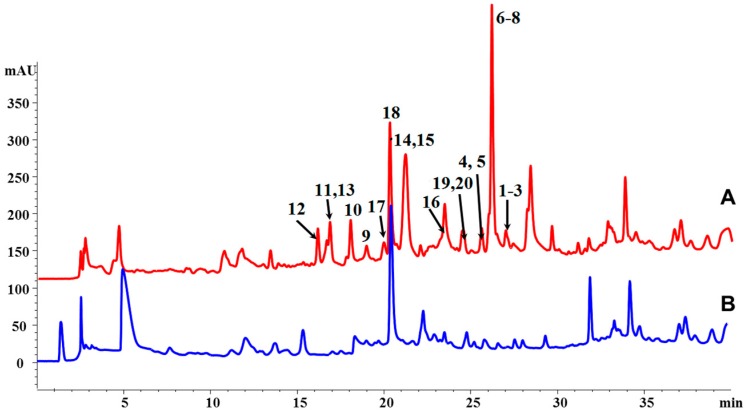
High Performance Liquid Chromatography (HPLC) analysis of the secondary metabolites of *P. chrysogenum* HLS111. (**A**) Test group (with valproic acid (VPA)). Peak numbers match the compound numbers in Figure 2 and Supplementary Materials Figure S1. (**B**) Control group.

**Figure 2:**
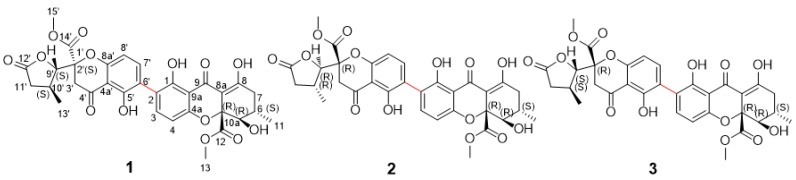
Chemical structures of compounds **1**–**3**.

The effects of HDAC inhibitors (valproic acid (VPA), suberoylanilide hydroxamic acid (SAHA), sodium butyrate (NaBu)) on the production of secondary metabolites of a sponge-associated fungus, *P. chrysogenum* HLS111, have been examined. As a result, the strain cultured with VPA produced more compounds than those of the control, and the contents of some compounds were much higher than those of the control (Figure 1). In this study, the differential parts of a crude extract of the test group attracted our attention, and its subsequent separation and purification provided three new tetrahydroxanthone—chromanone lactone heterdimers, designated chrysoxanthones A–C (**1**–**3**, Figure 2), and 17 known compounds (**4**–**20**, Supplementary Materials Figure S1). Herein, we report the isolation, structure elucidation, and biological activities of these compounds. Finally, the biosynthetic pathway of chrysoxanthones A–C was proposed.

## 2. Results and Discussion

### 2.1. Structure Elucidation of Compounds

The organic extract of *P. chrysogenum* HLS111 treated by VPA was purified by repeated silica-gel chromatography, reversed-phase chromatography, as well as semipreparative HPLC to yield compounds **1**–**20**. Chrysoxanthones A–C (**1**–**3**) were new isomerides that shared the common molecular formula of C_32_H_30_O_14_, determined by HRESIMS {*m*/*z* 639.1716 [M + H]^+^ (compound **1**), *m*/*z* 639.1712 [M + H]^+^ (compound **2**), *m*/*z* 639.1713 [M + H]^+^ (compound **3**), calcd for C_32_H_31_O_14_ 639.1714}, requiring 18 degrees of unsaturation. Compounds **1**–**3** have similar ultraviolet spectral properties *λ*_max_ (log ε) 248.2 (3.6), 335.5 (3.6) nm, indicating that they share related skeletons.

**Figure 3:**
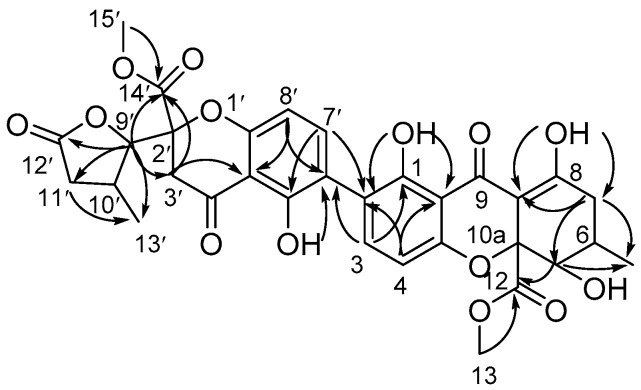
Key HMBC correlations of compounds **1**–**3**.

**Figure 4:**
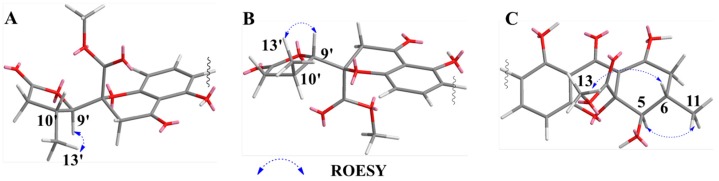
(**A**) Key Rotating Frame Overhauser Effect Spectroscopy (ROESY) correlations of β-methyl-γ-lactone moiety in compounds **1** and **2**; (**B**) key ROESY correlations of β-methyl-γ-lactone moiety in compound **3**; (**C**) key ROESY correlations of tetrahydroxanthone moiety in compounds **1**–**3**.

**Figure 5:**
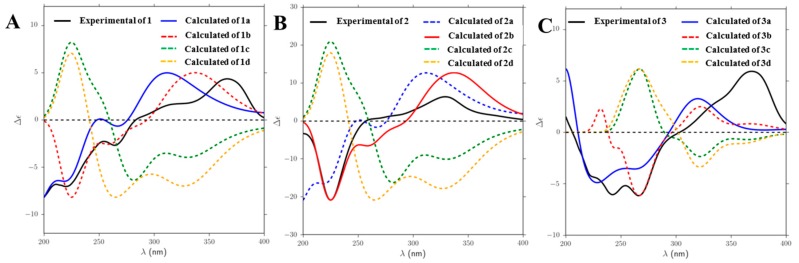
Experimentally measured and calculated electronic circular dichroism (ECD) spectra for compounds **1**–**3**.

Chrysoxanthones A (**1**) and B (**2**) were attained as a pale yellow powder. They showed identical ^1^H and ^13^C NMR spectra (Table 1, Supplementary Materials Figures S4, S5, S10, and S11), despite they were separated using a nonenantioselective method, presuming they were isomers but not enantiomers. By comparison of the NMR data (Table 1) with the structurally related compounds, they were identified as the tetrahydroxanthone–chromanone lactone heterdimers [6,7,8]. The HMBC correlations of H-3 to C-6′ and H-7 to C-2 indicated the monomers of compounds **1** and **2** were connected by a 2-6′ linkage (Figure 3, Supplementary Materials, Figures S6 and S12). The ^1^H and ^13^C NMR data (Table 1) of **1** and **2** were in agreement with those for blennolide J [6], which suggested they might have the same planar structures. The key coupling constants in ^1^H NMR spectra (Table 1, Supplementary Materials, Figures S4 and S10), the key Rotating Frame Overhauser Effect Spectroscopy (ROESY) correlations (Figure 4) and NMR data rules could prove the relative stereochemistry of **1** and **2**. The *trans*-configuration of H-5 and H-6 of the tetrahydroxanthone monomer was established by chemical shifts (compounds **1** and **2** δ_5-H/C_ 3.93/76.9, δ_6-H/C_ 2.42/29.2 vs. blennolide I δ_5-H/C_ 4.12/71.3, δ_6-H/C_ 2.11/28.5; *cis*-configuration) and the coupling constant (^3^*J*_H-5/H-6_ = 11.5 Hz) [6], as well as the ROESY correlation between H-5 (δ_H_ 3.93) and H-11 (δ_H_ 1.18). The ROESY correlation between H-13 (δ_H_ 3.68) and H-6 (δ_H_ 2.38) revealed the relative configurations of chiral carbons 5, 6, and 10a to be (5S, 6R, 10aS, or 5R, 6S, 10aR) (Figure 4C). Coupling constant ^3^*J*_H-9′/H-10′_ = 4.0 Hz established the *trans*-configuration of H-9′ and H-10′ of the *β*-methyl-*γ*-lactone moiety. Since the biaryl bond could rotate, it was ambiguous to determine the relative stereochemistry of carbon 2′ based on ROESY correlations. However, a summary of the NMR data [6,7,8] could provide some clues to identify the relative configurations of C-2′ and C-10′. When the difference between the H-3a’ and H-3b’ values was greater than 0.41 ppm, the relative configurations were proposed to be 2′S, 10′R or 2′R, 10′S, while when it was less than 0.19 ppm, the relative configuration was 2′S, 10′S or 2′R, 10′R. Thus, the relative configurations of **1** and **2** were the same as those of blennolide J [6], but the optical rotation data and CD spectra of compounds **1** and **2** were opposite to those of blennolide J (compound **1** ${[\alpha [}_{D}^{20}$ +30.8 (*c* 0.05, MeOH), compound **2** ${[\alpha [}_{D}^{20}$ +58.4 (*c* 0.15, MeOH), blennolide J ${[\alpha [}_{D}^{20}$ −93.5 (*c* 0.08, CHCl_3_)) [6], indicating the absolute configurations of two subunits of compounds **1** and **2** were the same or enantiotropic to those of blennolide J. Then, the absolute structures of compounds **1** and **2** were suggested to be (1a/2a (5R,6S,10aR,2′S,9′S,10′S); 1b/2b (5R,6S,10aR,2′R,9′R,10′R); 1d/2d (5S,6R,10aS,2′R,9′R,10′R)). The absolute configurations of compounds **1** and **2** were determined by comparing experimentally measured CD spectra with those calculated with the Time-Dependent Density Functional Theory (TD-DFT) method. The experimental electronic circular dichroism (ECD) spectrum of compounds **1** and **2** matched very well with the theoretical ECD spectrum for 1a(5R,6S,10aR,2′S,9′S,10′S) and 2b(5R,6S,10aR,2′R,9′R,10′R) (Figure 5A,B), respectively, confirming the absolute structures of **1** and **2**.

Compound **3** was also isolated as a pale yellow powder. Its NMR spectroscopic data were similar with that of compounds **1** and **2**, except the chemical shifts [H-3a’ (δ_H_ 3.05), H-3b’ (δ_H_ 3.21), H-9′ (δ_H_ 4.45); C-3′(δ_C_ 39.6), C-9′(δ_C_ 87.5)] moved to [H-3a’ (δ_H_ 3.10), H-3b’ (δ_H_ 3.51), H-9′ (δ_H_ 4.38); C-3′(δ_C_ 40.4), C-9′(δ_C_ 86.4)] (Table 1). The key HMBC correlations of compound **3** suggested the planar structure of **3** was identical with structures **1** and **2** (Figure 3). The obvious NMR differences might be induced by the change of the stereogenic centers of 2′, 9′, and 10′ in *β*-methyl-*γ*-lactone moiety. The *trans*-configuration of H-9′ and H-10′ (9′S,10′S or 9′R,10′R) of the *β*-methyl-*γ*-lactone moiety was uniform with compounds **1** and **2** by the chemical shifts (Table 1) and coupling constant (^3^*J*_H-9′/H-10′_= 3.5 Hz), together with the NOE correlations between H-13′ (δ_H_ 1.19) and H-9′ (δ_H_ 4.38) (Figure 4B). Therefore, epimerization at C-2′ led to the above NMR differences. The absolute configurations of chiral carbons 2′, 9′, and 10′ were assigned as (2′R,9′S,10′S or 2′S,9′R,10′R), which were in agreement with paecilin B [6]. Above all, the accordance of the experimentally measured CD spectra with the calculated ECD of 3a confirmed the absolute configuration of compound **3** was 5R,6S,10aR,2′R,9′S,10′S (Figure 5C).

The known compounds were identified as andrastins A (keto form, **4** and enol form, **5**) [9,10], secalonic acid A (**6**) [11,12,13,14], secalonic acid D (**7**) [14], secalonic acid F (**8**) [14,15], griseoxanthone C (**9**) [16], norlichexanthone (**10**) [16], 7-hydroxy-3-(2-hydroxypropyl)-5-methyl-isochromen-1-one (**11**) [17], 7-hydroxy-3,5-dimethyl-isochromen-1-one (**12**) [17], penicisimpin B (**13**) [18], meleagrin (**14**) [19], oxaline (**15**) [20], rcqueforcine C (**16**) [21], citreoindole (**17**) [22], haenamindole (**18**) [22], conidiogenone C (**19**) [23], and conidiogenone D (**20**) [23] by comparing their spectroscopic data with those reported in the literature (chemical structures in Supplementary Materials Figure S1). Moreover, the NMR data of andrastins A in keto form were reported the first time (Supplementary Materials Table S3).

### 2.2. Biological Assays

Chrysoxanthones A–C were assayed for antibacterial activities against *Staphylococcus epidermidis* (ATCC 12228, MSSE), *Staphylocccus aureus* (ATCC 29213, MSSA), *Bacillus subtilis* (ATCC 63501), *Enterococcus faecalis* (ATCC 29212, VSE), and *Escherichia coli* (ATCC 25922). Chrysoxanthones A–C exhibited the highest antibacterial activities against *B. subtilis* with minimum inhibitory concentration (MIC) values of 5–10 μg/mL and moderate activities against *S. epidermidis* and *S. aureus* with MICs of 10–80 μg/mL (Table 2). Meanwhile, the antitumor activities in vitro of chrysoxanthones A–C were evaluated against human multiform glioblastoma (U87 MG), human nonsmall cell lung cancer (NCI-H1650), human colonic carcinoma (HT29), human renal carcinoma (A498), and human leukemia (HL60) cell lines using the MTT method. Compared with secalonic acid D, the antitumor activities of chrysoxanthones A–C decreased significantly (Supplementary Materials Table S5), indicating that the *β*-methyl-*γ*-lactone ring reduced cytotoxicity, supporting that the tricyclic core was essential for bioactivities [6].

### 2.3. Plausible Biosynthetic Gene Cluster and Pathway of Chrysoxanthones A–C

**Scheme 1:**
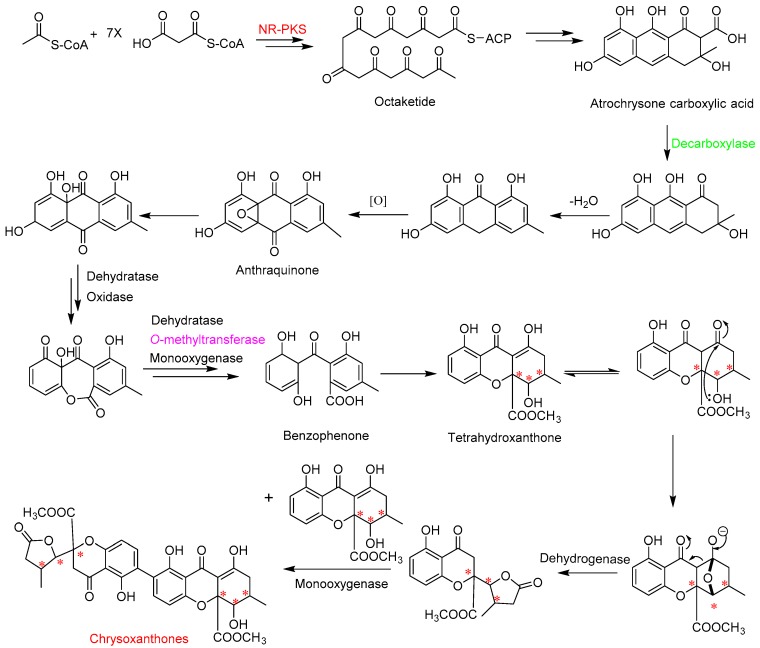
Plausible biosynthetic pathway of chrysoxanthones. Note: chiral carbons were marked with the red stars.

Based on the common tetrahydroxanthones moiety, we proposed that chrysoxanthones A–C shared the same biosynthetic gene cluster and pathway with those of secalonic acids. The biosynthetic gene cluster of secalonic acids in *Claviceps purpurea* has been confirmed by deleting gene *CPUR_05437*, which led to the abolishment of secalonic acid production [24]. In our effort to find the biosynthetic gene cluster, the gene *pc16g10750* of *P. chrysogenum* wisconsin 54-1255 showed the highest identity with the gene *CPUR_05437* (71%) by the NCBI BLAST. *Pc16g10750* putatively encodes an iterative nonreducing PKS (NR-PKS) and has a domain architecture of KS-AT-PT-ACP, as ascertained by NCBI Conserved Domains. In these special types of NR-PKSs lacking the thioesterase (TE) domain, polyketide is released from the PKS by a metallo-β-lactamase-type thioesterase (MβL-TE) [24]. The functions of the upstream and downstream genes were also analyzed by BLAST analysis, indicating the integrated gene cluster composed of 23 Open Reading Frames (ORFs) (Supplementary Materials, Figure S24). The gene cluster included one NR-PKS (*pc16g10750*), one *O*-methyltransferase (*pc16g10740*), two decarboxylases (*pc16g10730* and *pc16g10800*), two major facilitator superfamily proteins (*pc16g10710* and *pc16g10850*), two fungal specific transcription factors (*pc16g10640* and *pc16g10860*), ten oxidases or reductases, and five function-unknown genes (Supplementary Materials, Table S4). Finally, the plausible biosynthetic pathway of chrysoxanthones was depicted in Scheme 1. The route followed the sequence octaketide–anthraquinone–benzophenone–tetrahydroxanthone–*β*-methyl-*γ*-lactone–chrysoxanthone. As supposed in the Scheme 1, *β*-methyl-*γ*-lactone moiety was derived from the tetrahydroxanthone segment, while the absolute configurations of its chiral carbons (marked with the red stars) were not in accordance with that of the obtained tetrahydroxanthones. Hence, more tetrahydroxanthones with other absolute configurations may be discovered in the future.

## 3. Materials and Methods

### 3.1. General

Optical rotations were obtained on a JASCO P-2000 digital polarimeter (JASCO, Tokyo, Japan). UV spectra were recorded on a V-650 spectrometer (JASCO, Tokyo, Japan). Circular dichroism (CD) spectra were measured on a JASCO J-815 CD spectrometer (JASCO, Tokyo, Japan). IR spectra were collected on a Nicolet 5700 FT-IR microscope instrument (FT-IR microscope transmission) (Thermo Electron Corporation, Madison, WI, USA). ^1^H and ^13^C NMR and 2D NMR spectra were acquired at 500 and 125 MHz on a Bruker AVANCE 500-III instrument (Bruker Biospin Group, Karlsruhe, Germany) in chloroform-*d* with TMS as an internal reference, and chemical shifts were recorded as *δ* values. HR-ESI-MS data were measured using an Agilent 1100 LC/MSD Trap SL LC/MS/MS spectrometer (Agilent Technologies Ltd., Santa Clara, CA, USA). HPLC analysis of the secondary metabolites of the crude extracts was performed on an Agilent 1200 HPLC system (Agilent Technologies Ltd., Santa Clara, CA, USA), using a Cosmosil C18 column (5 μM, 4.6 × 150 mm, Nacalai Tesque, Inc., Kyoto, Japan). An HPLC system equipped with a Shimadzu LC-6AD pump, a Shimadzu SPD-20A prominence UV-VIS detector (Shimadzu Corporation, Kyoto, Japan), and an Agilent C18 column (5 μM, 250 × 9.6 mm) was used for semipreparative HPLC. Column chromatography was performed with silica gel (60–100 mesh, 200–300 mesh, Qingdao Marine Chemical Ltd., Qingdao, China), RP-18 (40–60 μM, GE Healthcare, Fairfield, CT, USA), and Sephadex LH-20 (18–110 M, GE healthcare, Fairfield, CT, USA).

### 3.2. Fungal Material and Fermentation

The fungal strain HLS111 was isolated from a *Gelliodes carnosa* sponge collected from Lingshui Bay, Hainan Province, China. It was identified as *P. chrysogenum* on the basis of morphology and internal transcribed spacer (ITS) sequence analysis. The sequence data have been deposited at GenBank as FJ770066 [25]. The fungus HLS111 was first cultivated on YPD agar plates (YPD: yeast extract 10 g, peptone 20 g, glucose 20 g, natural seawater 1 L, pH 7.0–7.2) at 28 °C for 3 days. Then, the mycelia were inoculated into 500 mL Erlenmeyer flasks, each containing 100 mL of liquid YPD medium. The flasks were incubated at 28 °C on a rotary shaker (220 rpm) for 3 days to prepare seed culture. The rice medium was used for solid fermentation, with each 500 mL Erlenmeyer flask containing 100 g rice and 100 mL natural seawater sterilized at 121 °C for 15 minutes. The test-group fermentation was carried out in 25 Erlenmeyer flasks with the HDAC inhibitor VPA at a final concentration of 10 μM, and the control-group fermentation was performed in 3 Erlenmeyer flasks. The flasks were inoculated with 20 mL of seed cultures and incubated at 28 °C for 30 days.

### 3.3. Extraction, HPLC Analysis, and Isolation

The fermented material was extracted with EtOAc (*v*/*v* 1:3) 3 times by ultrasound, and the organic solvent was evaporated to dryness under vacuum to afford the crude extract (the test group was 45 g and the control group was 4 g). The samples (10 mg/mL) of the test and control groups were analyzed with an Agilent 1200 HPLC system using a Cosmosil C18 column (4.6 × 150 mm, 5 μm) with a solvent gradient from 10% to 100% solvent B (solvent A: H_2_O, solvent B: CH_3_OH) over the course of 0 to 30 min and 100% solvent B over 30 to 40 min and UV detection at 230 nm at a flow rate of 1 mL/min.

The crude extract of the test group was first fractionated through vacuum liquid chromatography over a silica-gel column (8 × 100 cm, 1100 g), using a gradient elution with petroleum ether-EtOAc-CH_3_OH, (petroleum ether; petroleum ether-EtOAc = 100:1, 50:1, 20:1, 10:1, 5:1, 2:1, 1:1; EtOAc; CH_3_OH; *v*/*v*, each 2.5 L) to give 10 fractions (Fractions A–J). Fraction E (petroleum ether-EtOAc 10:1, 5.6 g) was further submitted to ODS CC (4 × 60 cm, 200 g) using a stepped gradient elution of CH_3_OH-H_2_O (20%, 50%, 80%, and 100%, *v*/*v*, each 1.5 L) to afford 4 subfractions E1–E4. Purification of the E4 subfraction (100% CH_3_OH, 800 mg) by semipreparative HPLC on an Agilent C18 column (9.6 × 250 mm, 5 μm) with 47% CH_3_CN in H_2_O as the mobile phase (flow rate 4 mL/min) afforded compounds **1**–**3** (compound **1**, 3.0 mg, t_R_ = 34.5 min; compound **2**, 8.3 mg, t_R_ = 26.7 min; compound **3**, 4.1 mg, t_R_ = 44.2 min). The isolation of the known compounds was shown in supporting information.

### 3.4. Spectroscopic Data

**Chrysoxanthone A** (**1**). Pale yellow powder; ${[\alpha [}_{D}^{20}$ +30.8 (c 0.05, MeOH); UV (MeOH) λ_max_ (log ε) 248.2 (3.6), 335.5 (3.6); CD (c 7.84 × 10^−4^ mol/L, MeOH), λ_max_ (Δε) 219 (−7.1), 264 (−2.7), 316.5 (1.6), 366.5 (4.3) nm; IR (KBr) ν_max_: 3519, 2959, 1792, 1741, 1613, 1566, 1433, 1360, 1213, 1060, 1022, 819 cm^−1^; ^1^H NMR (500 MHz, CDCl_3_) and ^13^C NMR (125 MHz, CDCl_3_) see Table 1; HR-ESI-MS (positive): *m*/*z* 639.1716 [M + H]^+^ (Calcd. for C_32_H_31_O_14_, 639.1714).

**ChrysoxanthoneB** (**2**). Pale yellow powder; ${[\alpha [}_{D}^{20}$ +58.4 (c 0.15, MeOH); UV (MeOH) λ_max_ (log ε) 248.2 (3.6), 335.5 (3.6); CD (*c* 7.84 × 10^−4^ mol/L, MeOH), λ_max_ (Δε) 225 (−20.9), 329 (6.4) nm; IR (KBr) ν_max_: 3511, 2958, 1792, 1741, 1613, 1566, 1434, 1359, 1211, 1060, 1023, 819 cm^−1^; ^1^H NMR (500 MHz, CDCl_3_) and ^13^C NMR (125 MHz, CDCl_3_) see Table 1; HR-ESI-MS (positive): *m*/*z* 639.1712 [M + H]^+^ (Calcd. for C_32_H_31_O_14_, 639.1714).

**ChrysoxanthoneC** (**3**). Pale yellow powder; ${[\alpha [}_{D}^{20}$ +33.2 (c 0.1, MeOH); UV (MeOH) λ_max_ (log ε) 248.2 (3.6), 335.5 (3.6); CD (*c* 7.84 × 10^−4^ mol/L, MeOH), λ_max_ (Δε) 242.5 (−6.1), 266 (−6.2), 369 (5.9) nm; IR (KBr) ν_max_: 3511, 2958, 1792, 1741, 1613, 1566, 1434, 1359, 1211, 1060, 1023, 819 cm^−1^; ^1^H NMR (500 MHz, CDCl_3_) and ^13^C NMR (125 MHz, CDCl_3_) see Table 1; HR-ESI-MS (positive): *m*/*z* 639.1713 [M + H]^+^ (Calcd. for C_32_H_31_O_14_, 639.1714).

### 3.5. ECD Calculation

TD-DFT calculations were executed with Gaussian 09 [26] (Gaussian, Inc., Pittsburgh, PA, USA). Conformational analysis was initially performed using Confab [27] at MMFF94 force field for all relative configurations of each compound. The conformers with lower energies (Boltzmann population of over 1%) were chosen for ECD calculations (Supplementary Materials, Table S1). The conformers were first optimized at PM6 using a semiempirical theory method and further at B3LYP/6-311G** in MeOH using the CPCM polarizable conductor calculation model (Supplementary Materials, Table S2). ECD calculations were conducted in MeOH using TD-DFT, and a total of 50 excited states were calculated. The BP86/6-311G** method was used for compounds **1** and **2**, while the TD-DFT theory level for compound **3**. The ECD and UV-Vis spectra were simulated in SpecDis [28] by overlapping Gaussian functions for each transition, and summed up by Boltzmann weights.

### 3.6. Biological Assay

Antibacterial and in vitro antitumor assays were performed for the isolated compounds (>90% purity).

#### 3.6.1. Antibacterial Activity

The antibacterial activities (represented by MIC values) of compounds **1**–**3** against 5 bacteria strains, *B. subitlis* ATCC 63501, *S. aureus* ATCC 29213, *S. epidermidis* ATCC 12228, *E. faecalis* ATCC 29212, and *E. coli* ATCC 25922, were evaluated by a 96-well plate-based assay [29]. The bacterial strains cultured in LB medium were collected at OD_600_ of 0.3~0.5, then diluted to OD_600_ of 5 × 10^−4^. Aliquots of this suspension (100 μL) were transferred into a 96-well plate. The tested compounds were added into the bacteria suspensions to give the desired concentration, and each concentration had triplicate values. The wells containing the same number of cells but no compounds were set as control groups. Ampicillin was used as the positive control. The 96-well-plates were then incubated at 37 °C for 18 h. The plates were then read using a microplate reader at 600 nm. MIC value was determined by the OD_600_ with the control without bacteria.

#### 3.6.2. Antitumor Activity

The antitumor activities (in vitro, represented by IC_50_ values) of compounds **1**–**3** against 5 tumor cell lines, human multiform glioblastoma (U87 MG), human nonsmall cell lung cancer (NCI-H1650), human colonic carcinoma (HT29), human renal carcinoma (A498), and human leukemia (HL60), were evaluated with the MTT method, and secalonic acid D was used as the positive control. The detailed methodology for biological testing has already been described in a previous report [30].

## 4. Conclusions

Three new compounds, chrysoxanthones A–C (**1**–**3**), along with 17 known compounds (**4**–**20**) were isolated from the strain *P. chrysogenum* HLS111 cultivated with the histone-deacetylase inhibitor VPA. Our present findings revealed that histone-deacetylase inhibitors could activate diversified compound production or increase the yields of fungal secondary metabolites. Considering the instability and high cost of inhibitors, further deletion of genes related to epigenetic modifications still needs to be conducted.

## Figures and Tables

**Table 1:** ^1^H (500 MHz) and ^13^C NMR (125 MHz) data of compounds **1**–**3** in CDCl_3._

Position	1 and 2		3	
	*δ*_H_, mult, *J* in Hz	*δ*_C_	*δ*_H_, mult, *J* in Hz	*δ*_C_
1		159.3		159.3
2		117.7		117.8
3	7.46, d, 8.5	140.1	7.46, d, 8.5	140.2
4	6.63, d, 8.5	107.5	6.62, d, 8.5	107.6
4a		158.4		158.3
5	3.93, dd, 11.5, 2.5	76.9	3.93, dd, 11.0, 1.5	76.9
6	2.42, m	29.2	2.42, m	29.2
7a	2.32, dd, 19.0, 10.5	36.2	2.31, dd, 19.0, 10.5	36.3
7b	2.74, dd, 19.0, 6.5		2.74, dd, 19.0, 6.5	
8		177.6		177.6
8a		101.5		101.5
9		187.1		187.1
9a		106.8		106.8
10a		84.7		84.7
11	1.18, d, 6.5	18.0	1.17, d, 6.5	18.0
12		170.2		170.2
13	3.73, s	53.3	3.73, s	53.3
1-OH	11.72, s		11.72, s	
5-OH	2.78, d, 2.5		2.80, br s	
8-OH	13.76, s		13.78, s	
2′		84.2		84.3
3a’	3.05, d, 17.0	39.6	3.10, d, 17.0	40.4
3b’	3.21, d, 17.0		3.51, d, 17.0	
4′		194.0		195.0
4a’		107.6		107.3
5′		159.2		159.1
6′		118.1		118.0
7′	7.52, d, 8.5	141.3	7.51, d, 8.5	141.1
8′	6.62, d, 8.5	107.3	6.57, d, 8.5	107.0
8a’		158.6		158.6
9′	4.45, d, 4.0	87.5	4.38, d, 3.5	86.4
10′	2.84, m	30.0	2.88, m	29.6
11a’	2.92, dd, 17.5, 9.0	36.0	3.03, dd, 18.0,9.0	36.2
11b’	2.23, dd, 17.5, 4.0		2.23, dd, 18.0,4.0	
12′		175.2		175.5
13′	1.29, d, 7.0	20.9	1.19, d, 7.0	20.6
14′		168.8		169.0
15′	3.77, s	53.7	3.78, s	53.7
5′-OH	11.89, s		11.92, s	

**Table 2:** Minimum inhibitory concentrations (MIC, μg/mL) of compounds **1**–**3**.

Compounds	MICs (μg/mL)				
	*Staphylococcus epidermidis* ATCC 12228	*Staphylococcus aureus* ATCC 29213	*Bacillus subtilis* ATCC 63501	*Enterococcus faecalis* ATCC 29212	*Escherichia coli* ATCC25922
**1**	---	---	5	---	>100
**2**	10	20	5	>100	>100
**3**	20	80	10	>100	>100
**Ampicillin**	6.25	6.25	3.12	6.25	25

Note: “---” represents untested.

## Supplementary Materials

The following are available online at http://www.mdpi.com/1660-3397/16/10/357/s1: Figure S1: Chemical structures of the known compounds **4**–**20**; Figure S2: (+)-HRESIMS spectrum of chrysoxanthone A (**1**); Figure S3: IR spectrum of chrysoxanthone A (**1**); Figure S4: ^1^H NMR spectrum of chrysoxanthone A (**1**) in CDCl_3_; Figure S5: ^13^C NMR spectrum of chrysoxanthone A (**1**) in CDCl_3_; Figure S6: HMBC spectrum of chrysoxanthone A (**1**) in CDCl_3_; Figure S7: ROESY spectrum of chrysoxanthone A (**1**) in CDCl_3_; Figure S8: (+)-HRESIMS spectrum of chrysoxanthone B (**2**); Figure S9: IR spectrum of chrysoxanthone B (**2**); Figure S10: ^1^H NMR spectrum of chrysoxanthone B (**2**) in CDCl_3_; Figure S11: ^13^C NMR spectrum of chrysoxanthone B (**2**) in CDCl_3_; Figure S12: HMBC spectrum of chrysoxanthone B (**2**) in CDCl_3_; Figure S13: ROESY spectrum of chrysoxanthone B (**2**) in CDCl_3_; Figure S14: (+)-HRESIMS spectrum of chrysoxanthone C (**3**); Figure S15: IR spectrum of chrysoxanthone C (**3**); Figure S16: ^1^H NMR spectrum of chrysoxanthone C (**3**) in CDCl_3_; Figure S17: ^13^C NMR spectrum of chrysoxanthone C (**3**) in CDCl_3_; Figure S18: HMBC spectrum of chrysoxanthone C (**3**) in CDCl_3_; Figure S19: ROESY spectrum of chrysoxanthone C (**3**) in CDCl_3_; Table S1: Energies of dominative conformers of compounds **1**–**3** at MMFF94 force field; Table S2: Energies of the conformers of compounds **1**–**3** at B3LYP/6-311G** in methanol; Table S3: ^1^H (500 MHz) and ^13^C NMR (125 MHz) spectroscopic data (δ in ppm, J in Hz) for compound **4** in CDCl_3_; Figure S20: ^1^H NMR spectrum of andrastin A (**4**, in keto form) in CDCl_3_; Figure S21: ^13^ C NMR spectrum of andrastin A (**4**, in keto form) in CDCl_3_; Figure S22: HSQC spectrum of andrastin A (**4**, in keto form) in CDCl_3_; Figure S23: HMBC spectrum of andrastin A (**4**, in keto form) in CDCl_3_; Figure S24: Proposed biosynthetic gene cluster (*pc16g10640-pc16g10860*) of compounds **1**–**3**; Table S4: Putative ORFs and predicted functions of gene cluster *pc16g10640*-*pc16g10860*; Table S5: Antitumor activities (in vitro IC_50_, μM) of compounds **1**–**3**.
