# Supplementary material for: Chrysoxanthones A–C, Three New Xanthone–Chromanone Heterdimers from Sponge-Associated Penicillium chrysogenum HLS111 Treated with Histone Deacetylase Inhibitor
Source: Mar Drugs. 2018 Oct 1;16(10):357. doi: 10.3390/md16100357 (PMC6213349; doi:10.3390/md16100357)
Supplement: Supplementary file 1 [file marinedrugs-16-00357-s001.pdf]

Supplementary Information

1. **Figure S1.** Chemical structures of known compounds **4–20.**

2. **Figure S2.** The (+)-HRESIMS spectrum of chrysoxanthone A (**1**)

3. **Figure S3.** IR spectrum of chrysoxanthone A (**1**)

4. **Figure S4.** ^{1}H NMR spectrum of chrysoxanthone A (**1**) in $CDCl_3$

5. **Figure S5.** ^{13}C NMR spectrum of chrysoxanthone A (**1**) in $CDCl_3$

6. **Figure S6.** HMBC spectrum of chrysoxanthone A (**1**) in $CDCl_3$

7. **Figure S7.** ROESY spectrum of chrysoxanthone A (**1**) in $CDCl_3$

8. **Figure S8.** The (+)-HRESIMS spectrum of chrysoxanthone B (**2**)

9. **Figure S9.** IR spectrum of chrysoxanthone B (**2**)

10. **Figure S10.** ^{1}H NMR spectrum of chrysoxanthone B (**2**) in $CDCl_3$

11. **Figure S11.** ^{13}C NMR spectrum of chrysoxanthone B (**2**) in $CDCl_3$

12. **Figure S12.** HMBC spectrum of chrysoxanthone B (**2**) in $CDCl_3$

13. **Figure S13.** ROESY spectrum of chrysoxanthone B (**2**) in $CDCl_3$

14. **Figure S14.** The (+)-HRESIMS spectrum of chrysoxanthone C (**3**)

15. **Figure S15.** IR spectrum of chrysoxanthone C (**3**)

16. **Figure S16.** ^{1}H NMR spectrum of chrysoxanthone C (**3**) in $CDCl_3$

17. **Figure S17.** ^{13}C NMR spectrum of chrysoxanthone C (**3**) in $CDCl_3$

18. **Figure S18.** HMBC spectrum of chrysoxanthone C (**3**) in $CDCl_3$

19. **Figure S19.** ROESY spectrum of chrysoxanthone C (**3**) in $CDCl_3$

20. **Table S1.** Energies of dominative conformers of compounds **1–3** at MMFF94 force field.

21. **Table S2.** Energies of conformers of compounds **1–3** at B3LYP/6-311G** in methanol.

22. **Table S3.** ^{1}H (500 MHz) and ^{13}C NMR (125 MHz) spectroscopic data (δ in ppm, J in Hz) for compound **4** in $CDCl_3$

23. **Figure S20.** ^{1}H NMR spectrum of andrastin A (**4, in keto form**) in $CDCl_3$

24. **Figure S21.** ^{13} C NMR spectrum of andrastin A (**4, in keto form**) in $CDCl_3$

25. **Figure S22.** HSQC spectrum of andrastin A (**4, in keto form**) in $CDCl_3$

26. **Figure S23.** HMBC spectrum of andrastin A (**4, in keto form**) in $CDCl_3$

27. **Figure S24.** Proposed biosynthetic gene cluster (*pc16g10640- pc16g10860*) of compounds **1–3**

28. **Table S4.** Putative ORFs and predicted functions of gene cluster *pc16g10640-pc16g10860*

29. **Table S5.** Antitumor activities (in vitro IC_{50}, μM) of compounds **1–3**

Isolation of the Known Compounds. Fraction E3 (80% CH_3OH-H_2O, 760 mg) was separated on a semipreparative HPLC with an Agilent C_{18} column (9.6 × 250 mm, 5 μm) with 60% CH_3OH in H_2O as the mobile phase (flow rate 4 mL/min) to afford compounds **4–8** (compound **4,** 5.6 mg, t_{R5} = 35.2 min; compound **5** 2.3 mg, t_{R6} = 38.9 min; compound **6,** 28.2 mg, t_{R7} = 42.8 min; compound **7,** 25.3 mg, t_{R8} = 44.5 min; compound **8,** 24.1 mg, t_{R9} = 48.7 min). Fraction F (petroleum ether-EtOAc 5:1, 1.5 g) was chromatographed on Sephadex LH-20 (2.5 × 120 cm, 100 g) with $CHCl_3$-CH_3OH (1:10, *v/v,* each 20 mL) as eluent. Fractions were examined by HPLC and combined to fraction F1–F3. Purification of fraction F1 (180 mg) by semipreparative HPLC (45% CH_3OH/H_2O, 4 mL/min) afforded compound **17–18** (compound **17,** 6.2 mg, t_{R18} = 32.2 min; compound **18,** 4.2 mg t_{R19} = 34.5 min). Then, fraction F2 (340 mg) was subjected to the semipreparative HPLC (52% CH_3OH/H_2O, 4 mL/min) to provide compounds **14–16** (compound **14,** 6.6 mg, t_{R15} = 33.8 min; compound **15,** 6.9 mg, t_{R16} = 38.7 min; compound **16,** 5.4 mg t_{R17} = 41.6 min). Purification of fraction F3 (220 mg) by semipreparative HPLC (55% CH_3OH/H_2O, 4 mL/min) afforded compounds **19–20** (compound **19,** 5.2 mg, t_{R20} = 28.8 min; compound **20,** 4.7 mg, t_{R21} = 34.7 min). Fraction G (petroleum ether-EtOAc 2:1, 4.8 g) was separated by ODS CC (4 × 60 cm, 200 g), using a stepped gradient elution of CH_3OH-H_2O (40%, 65%, 85%, and 100%, *v/v,* each 1.5 L) to afford four subfractions, G1–G4. Fraction G1 (195 mg) was subjected to the semipreparative HPLC (35% CH_3OH/H_2O, 4 mL/min) to provide compounds **11–13** (compound **11,** 3.5 mg, t_{R12} = 38.1min; compound **12,** 4.3 mg, t_{R13} = 35.9 min; compound **13,** 3.9 mg t_{R14} = 39.6 min). Purification of fraction G2 (110 mg) by semipreparative HPLC (40% CH_3OH/H_2O, 4 mL/min) was performed to give compounds **9–10** (compound **9,** 4.8 mg, t_{R10} = 30.4 min; compound **10,** 6.1 mg, t_{R11} = 35.4 min).

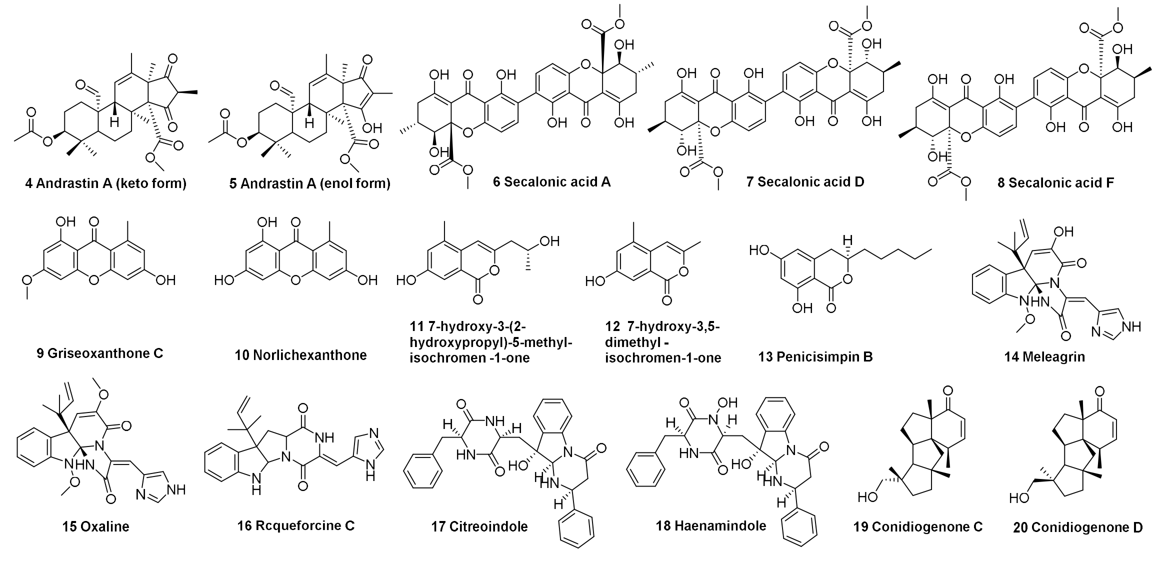

Figure S1. Chemical structures of known compounds **4–20.**

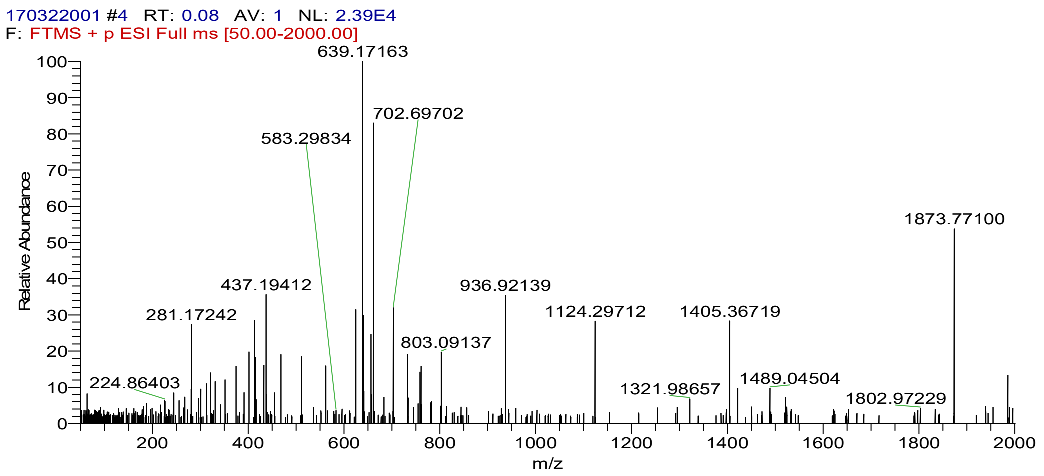

Figure S2. The (+)-HRESIMS spectrum of chrysoxanthone A (**1**)

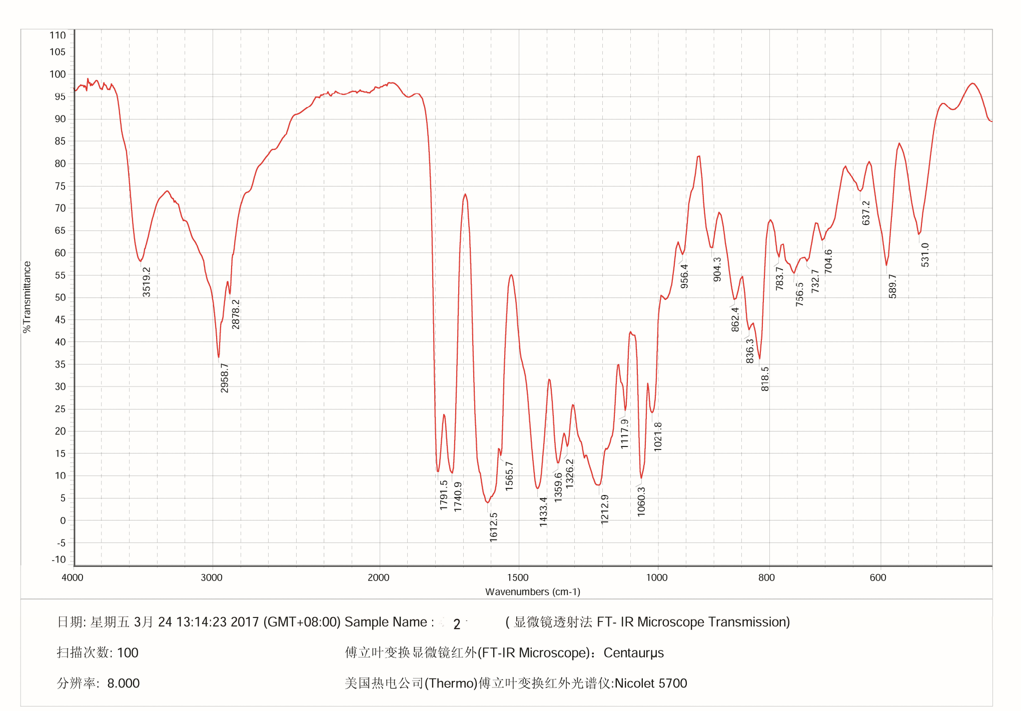

Figure S3. IR spectrum of chrysoxanthone A (**1**)

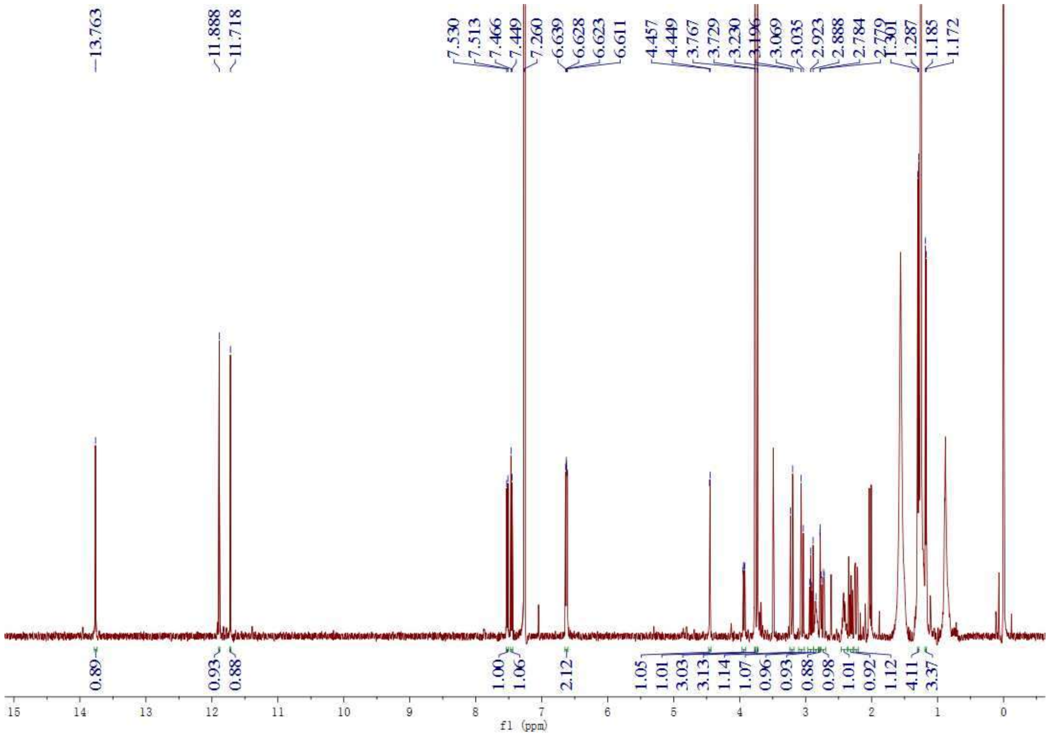

Figure S4. ^{1}H NMR spectrum of chrysoxanthone A (**1**) in $CDCl_3$

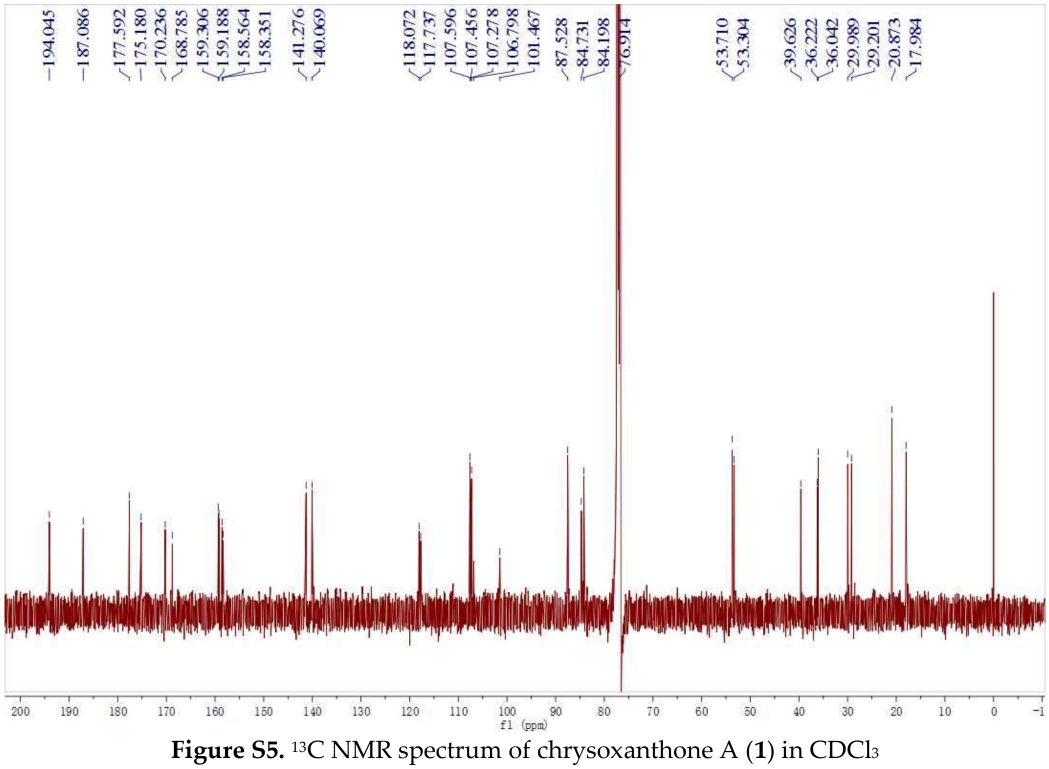

Figure S5. ^{13}C NMR spectrum of chrysoxanthone A (**1**) in $CDCl_3$

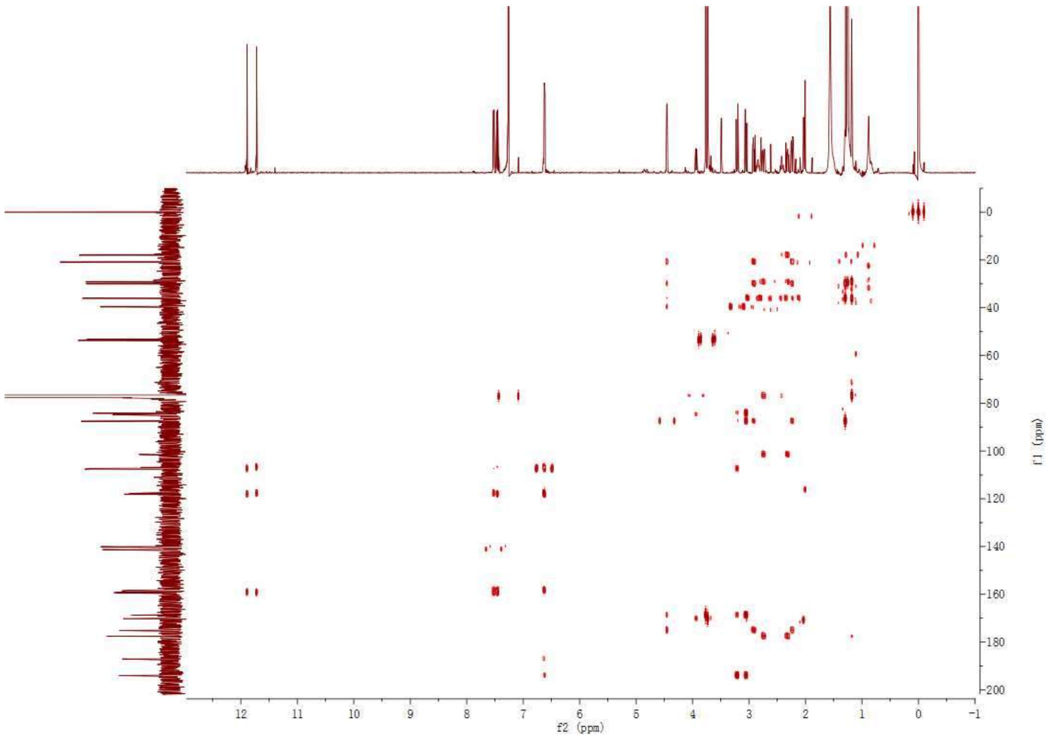

Figure S6. HMBC spectrum of chrysoxanthone A (**1**) in $CDCl_3$.

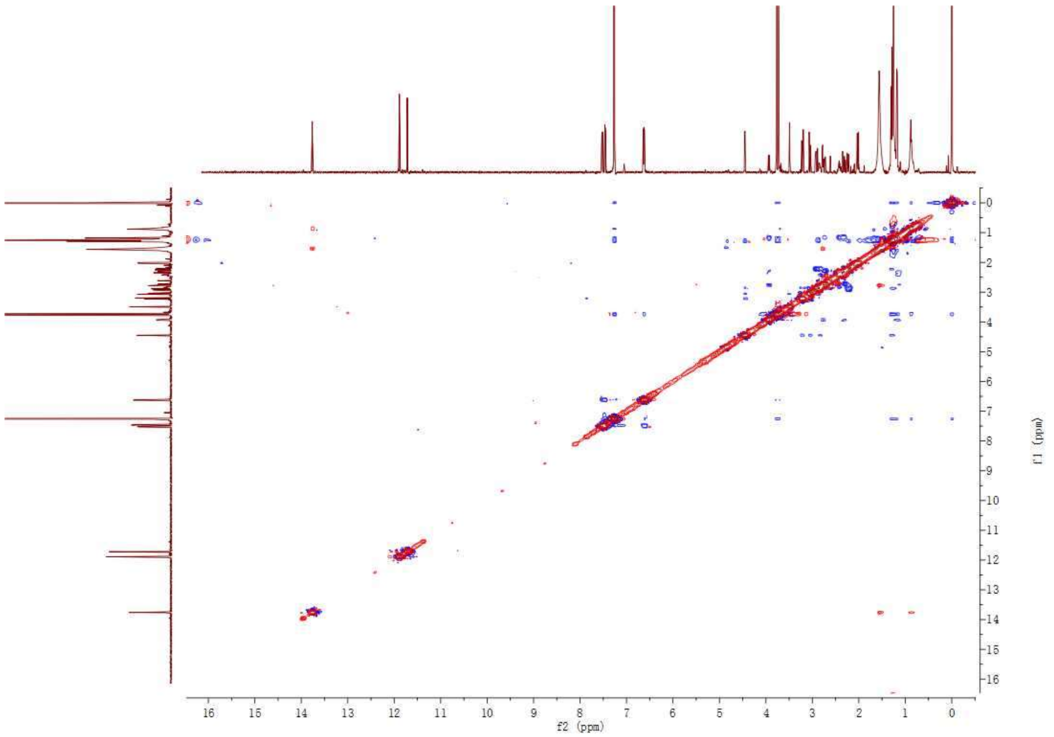

Figure S7. ROESY spectrum of chrysoxanthone A (**1**) in $CDCl_3$.

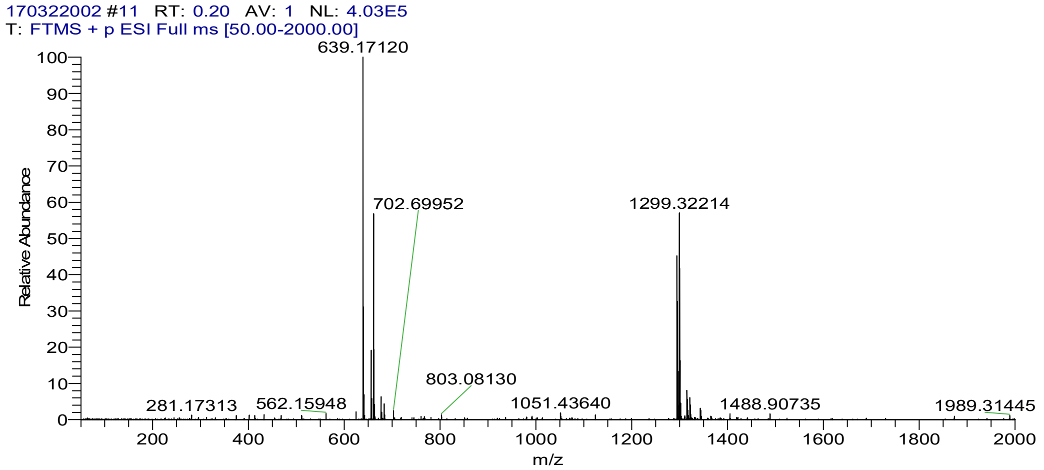

Figure S8. The (+)-HRESIMS spectrum of chrysoxanthone B (**2**)

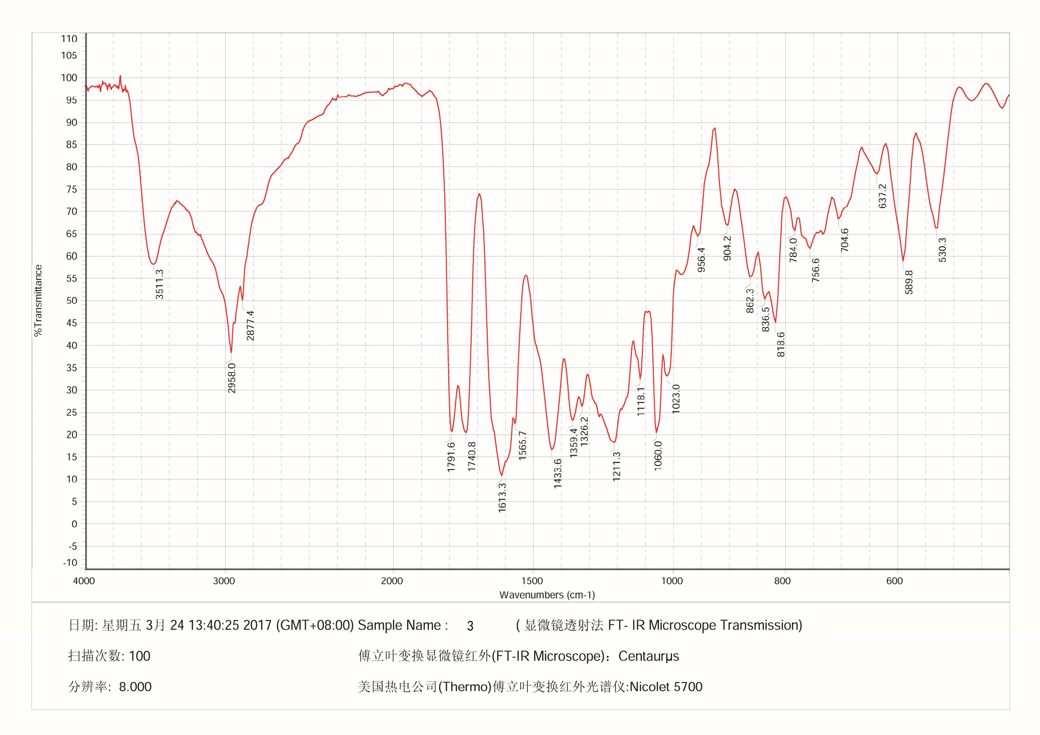

Figure S9. IR spectrum of chrysoxanthone B (**2**).

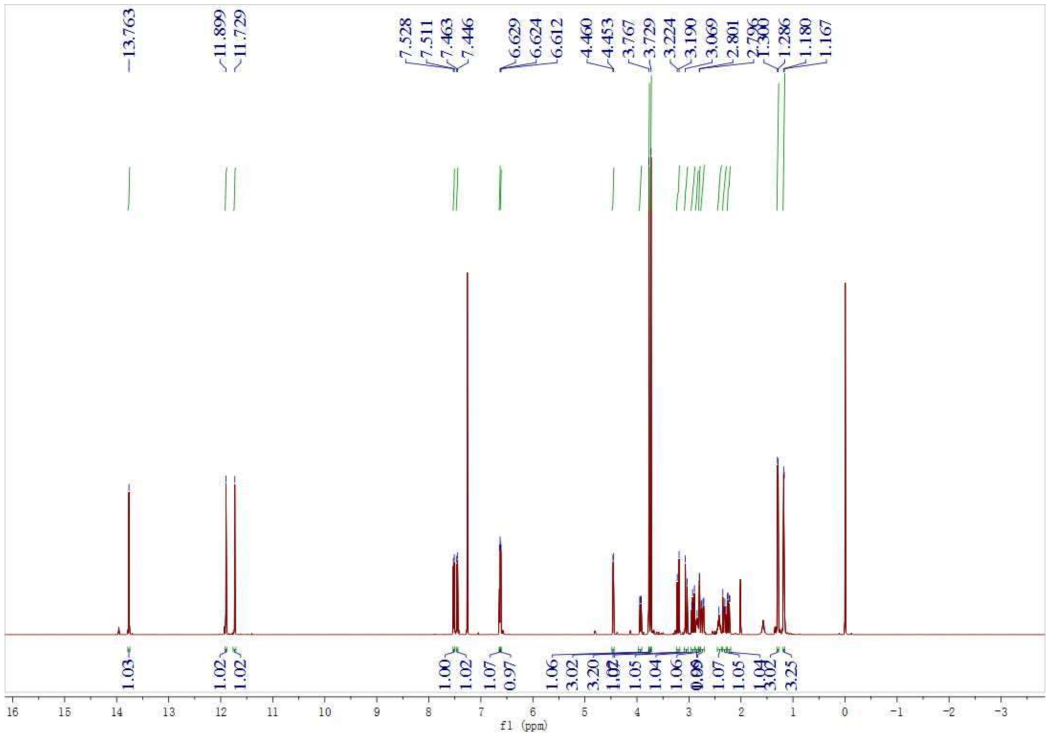

Figure S10. ^{1}H NMR spectrum of chrysoxanthone B (**2**) in $CDCl_3$.

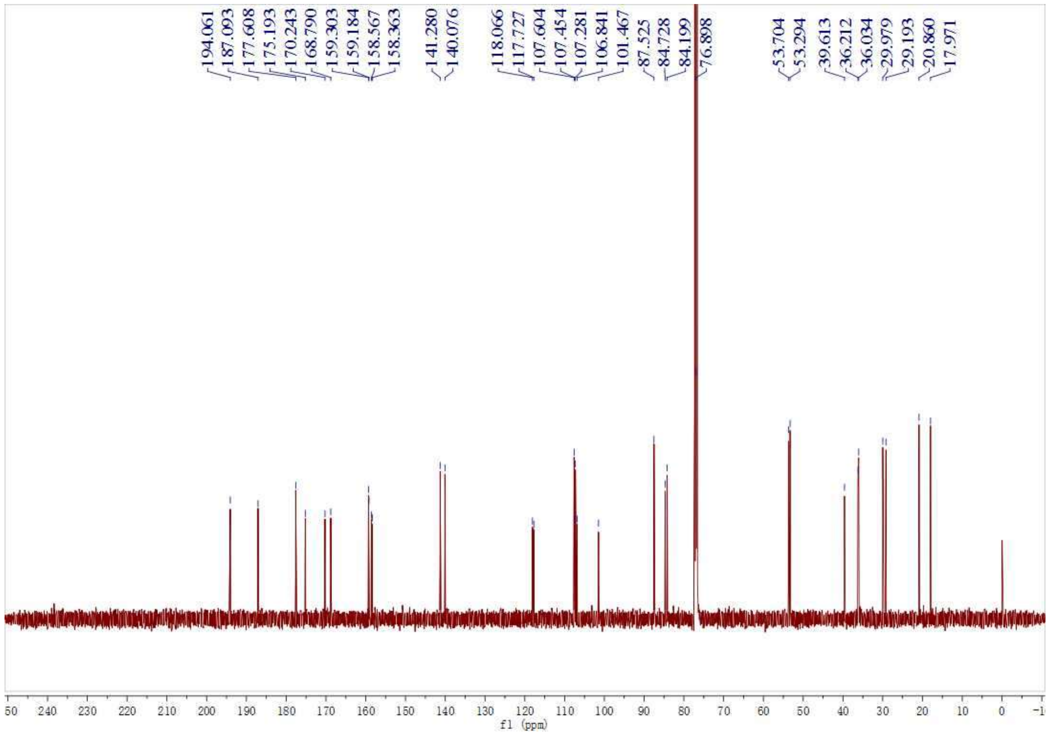

Figure S11. ^{13}C NMR spectrum of chrysoxanthone B (**2**) in $CDCl_3$

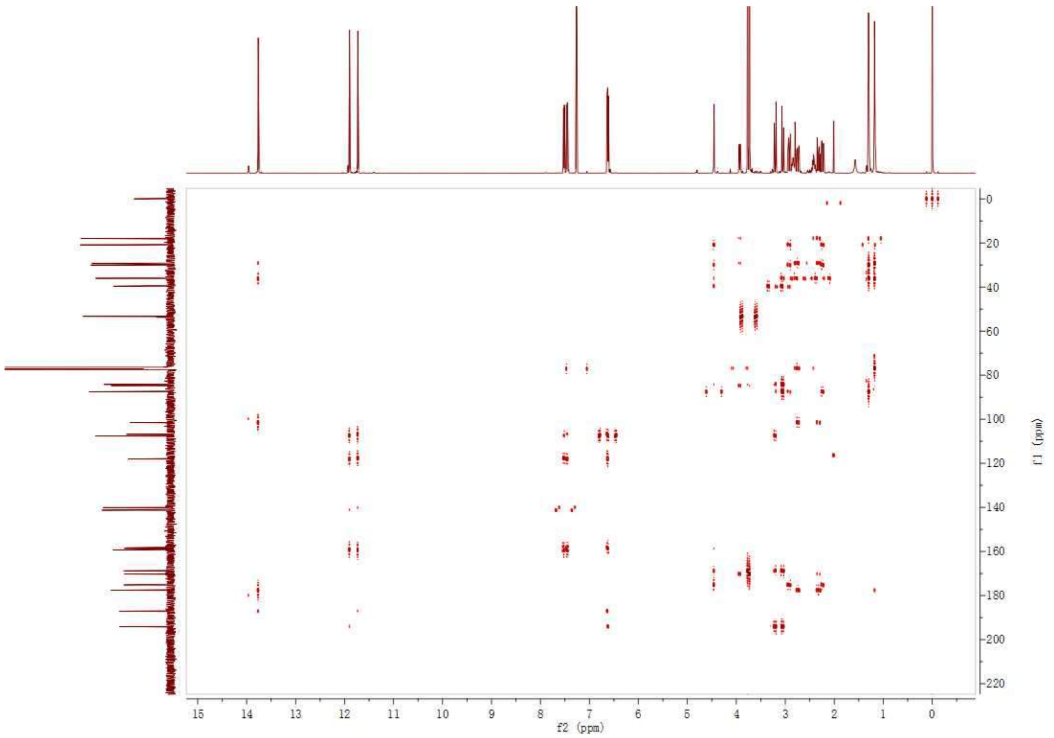

Figure S12. HMBC spectrum of chrysoxanthone B (**2**) in $CDCl_3$.

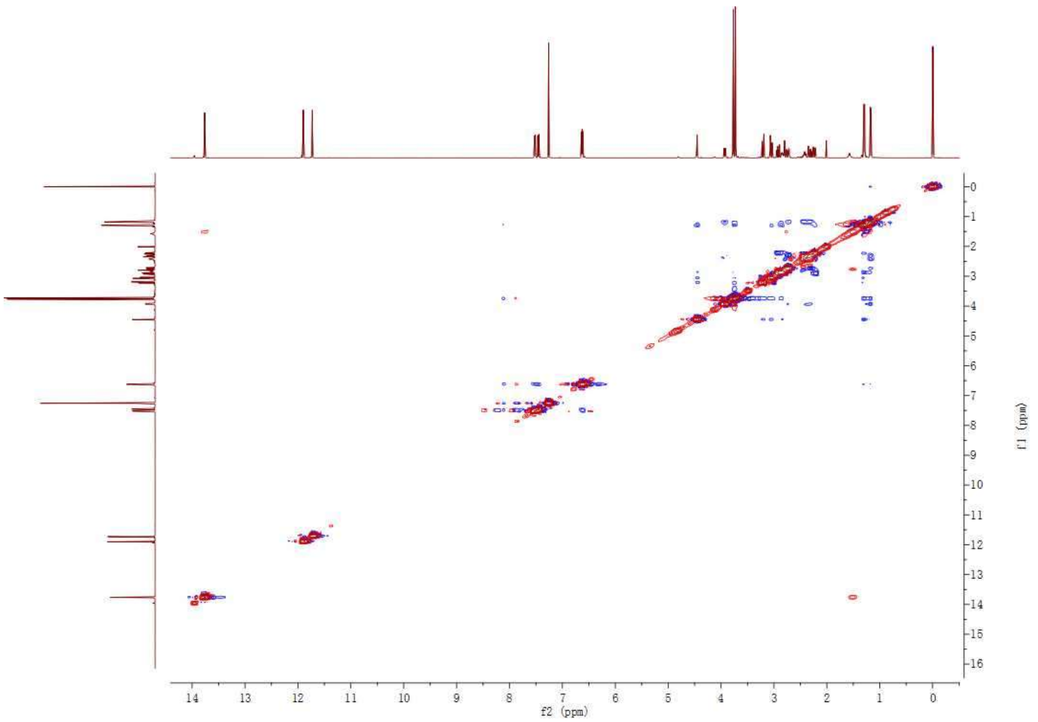

Figure S13. ROESY spectrum of chrysoxanthone B (**2**) in $CDCl_3$.

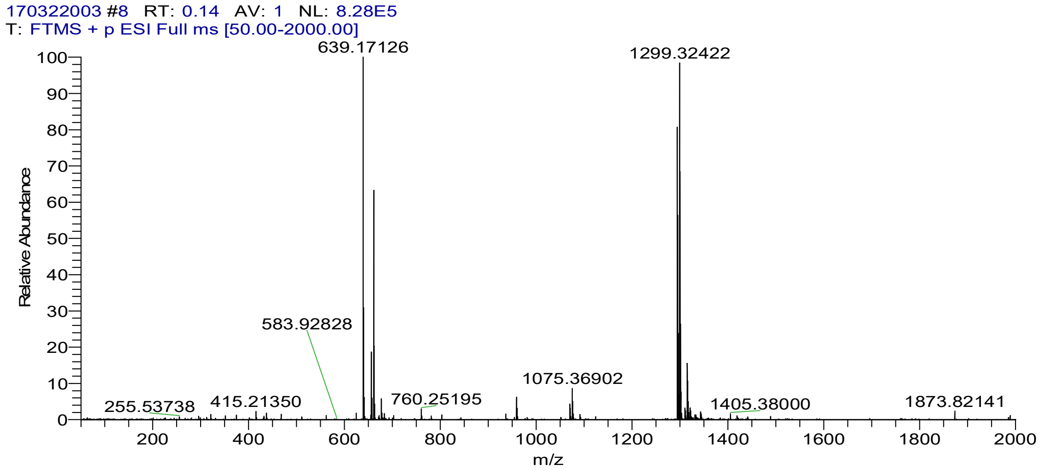

Figure S14. The (+)-HRESIMS spectrum of chrysoxanthone C (**3**).

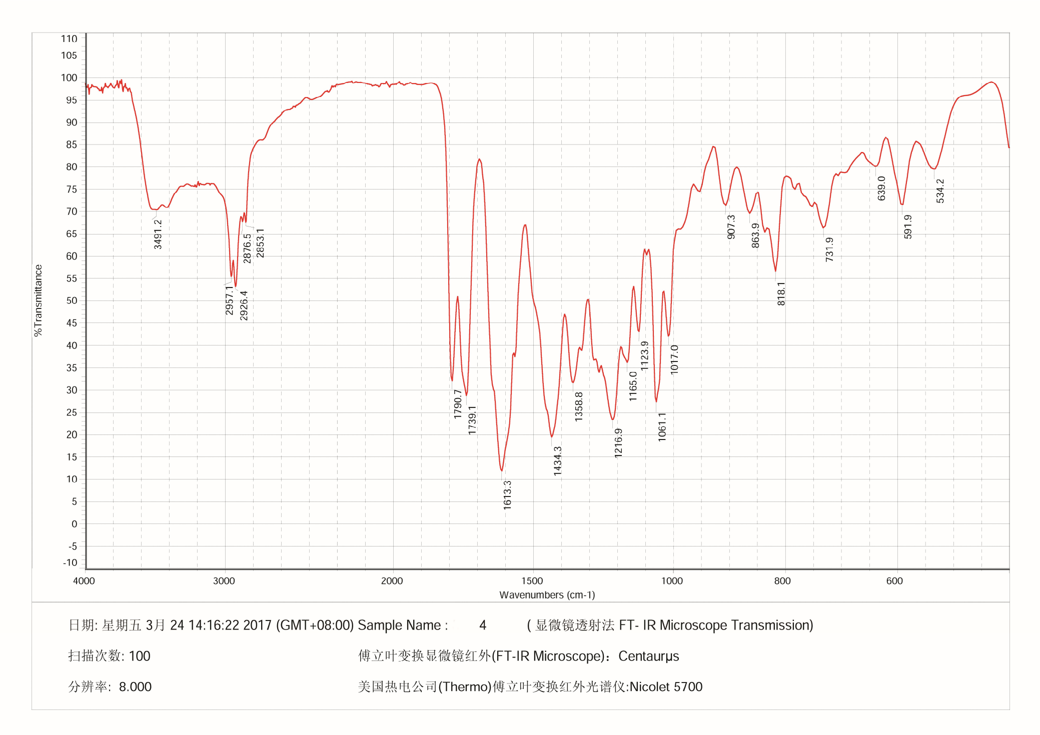

Figure S15. IR spectrum of chrysoxanthone C (**3**).

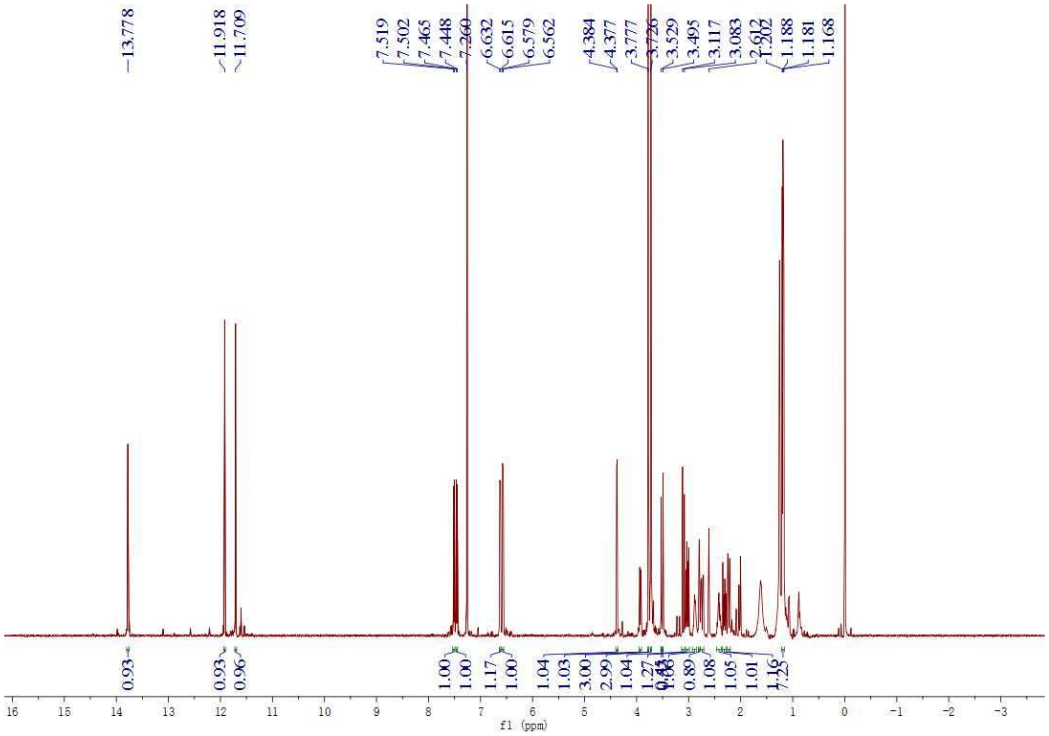

Figure S16. ^{1}H NMR spectrum of chrysoxanthone C (**3**) in $CDCl_3$.

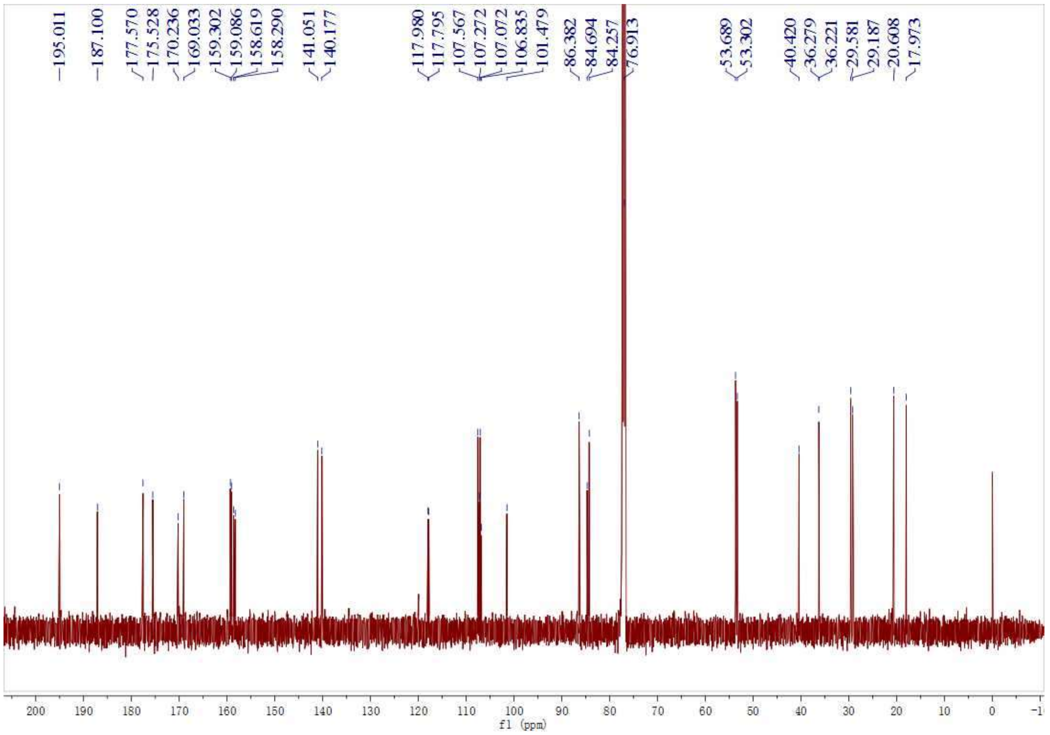

Figure S17. ^{13}C NMR spectrum of chrysoxanthone C (**3**) in $CDCl_3$.

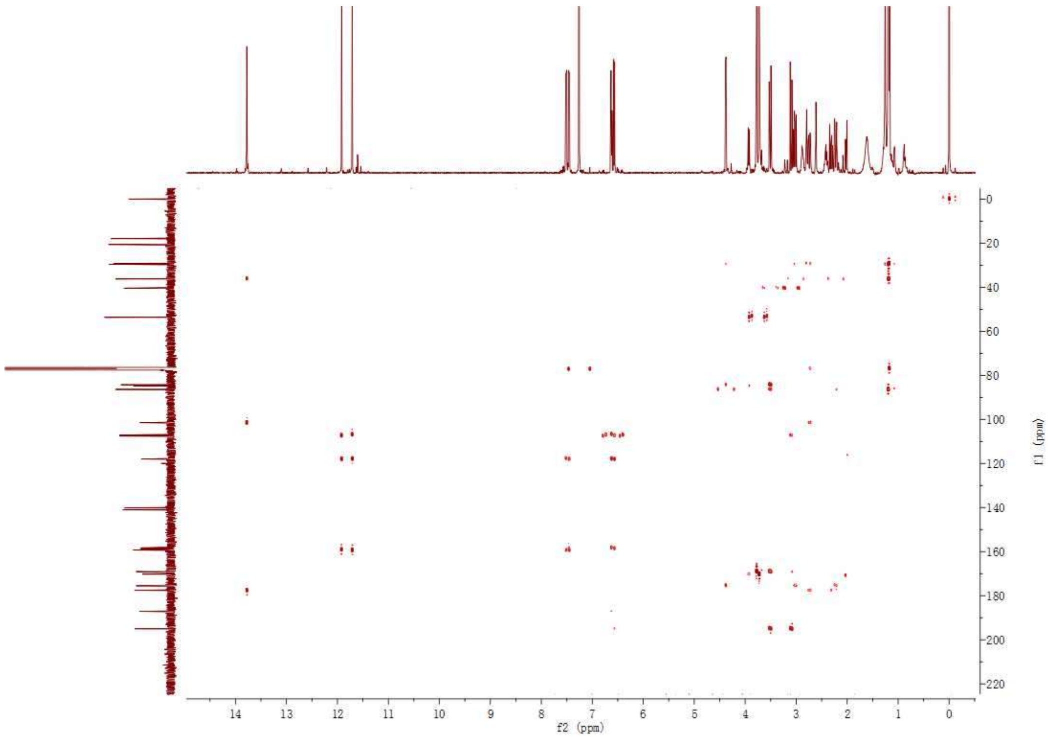

Figure S18. HMBC spectrum of chrysoxanthone C (**3**) in $CDCl_3$.

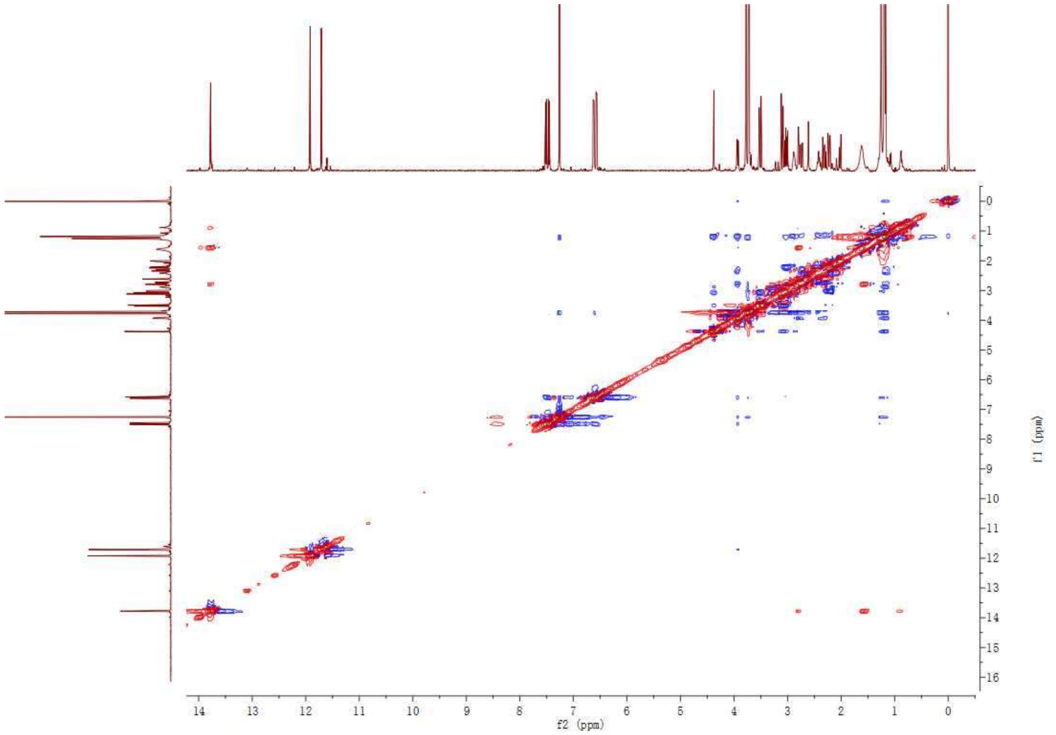

Figure S19. ROESY spectrum of chrysoxanthone C (**3**) in $CDCl_3$.

Table S1. Energies of dominative conformers of compounds **1–3** at MMFF94 force field.

Configuration	Conformer	Energy (kcal/mol)	Population (%)
1a/2a	1	154.07	88.60
	2	155.66	6.04
	3	156.15	2.64
	4	156.47	1.53
1b/2b	1	156.25	60.04
	2	156.49	39.84
1c/2c	1	155.09	92.11
	2	156.63	6.83
1d/2d	1	152.51	70.93
	2	153.05	28.96
3a	1	157.52	96.14
	2	159.72	2.32
	3	160.01	1.43
3b	1	155.77	95.99
	2	157.65	3.99
3c	1	153.31	91.33
	2	154.71	8.60
3d	1	151.34	53.20
	2	151.58	35.84
	3	152.28	10.96

Table S2. Energies of conformers of compounds **1 ~ 3** at B3LYP/6-311G** in methanol.

Configuration	Conformation	Structure	E (Hartree)	E (kcal/mol)	Population (%)
1a/2a	1	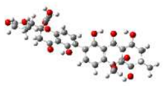	–2291.321942	–1437826.215	1.73
1a/2a	2	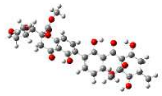	–2291.321588	–1437825.993	1.19
1a/2a	3	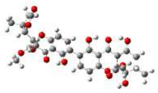	–2291.324721	–1437827.959	32.92
1a/2a	4	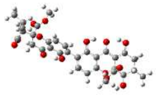	–2291.325351	–1437828.354	64.17
1b/2b	1	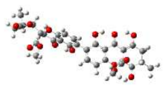	–2291.321538	–1437825.962	62.09
1b/2b	2	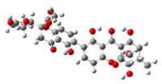	–2291.321073	–1437825.67	37.91

1c/2c	1	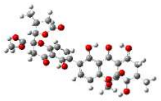	–2291.320084	–1437825.049	97.46
1c/2c	2	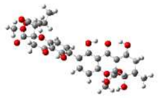	–2291.316644	–1437822.891	2.54
1d/2d	1	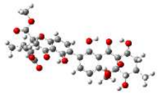	–2291.320168	–1437825.102	99.35
1d/2d	2	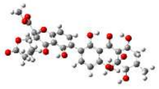	–2291.315428	–1437822.127	0.65
3a	1	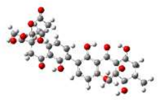	–2291.287827	–1437804.807	20.11
3a	2	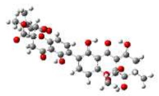	–2291.288303	–1437805.106	33.31
3a	3	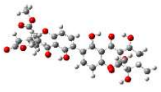	–2291.288619	–1437805.304	46.58

3b	1	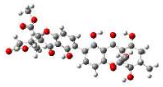	–2291.28883	–1437805.437	57.64
3b	2	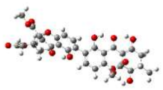	–2291.28854	–1437805.255	42.36
3c	1	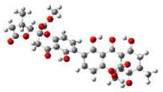	–2291.292728	–1437807.883	7.98
3c	2	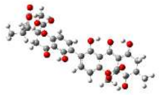	–2291.295034	–1437809.33	92.02
3d	1	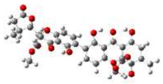	–2291.292081	–1437807.477	3.89
3d	2	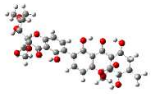	–2291.295046	–1437809.338	90.22
3d	3	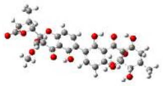	–2291.292472	–1437807.722	5.89

Table S3. ^{1}H (500 MHz) and ^{13}C NMR (125 MHz) spectroscopic data (δ in ppm, *J* in Hz) for compound **4** in $CDCl_3$

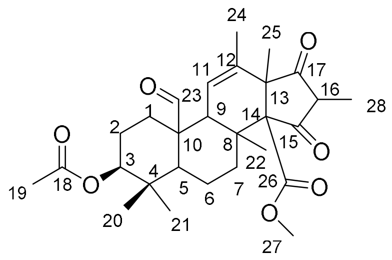

Position	δ_H, mult,*J* in Hz	δ_C	Position	δ_H, mult,*J* in Hz	δ_C
1a	2.35,ddd,12.5,3.5,3.5	27.7	14		72.3
1b	1.98,ddd,14.0,12.5,4.0		15		210.5
2	1.60,m	23.4	16	3.22,q,7.0	50.8
3	4.65,t,2.5	77.2	17		208.9
4		37.0	18		170.7
5	1.78,dd,10.8,3.2	47.9	19	2.10,s	21.4
6	1.60,m	16.7	20	0.88,s	21.2
7a	2.73,ddd,13.5,13.0,4.0	30.9	21	0.94.s	26.5
7b	2.42,ddd,13.5, 3.5,3.0		22	1.17,s	19.0
8		39.4	23	10.12,s	204.4
9	2.12,d,2.0	53.8	24	1.66,brs	19.0
10		52.3	25	1.24,s	15.9
11	5.72,s	124.7	26		168.0
12		134.3	27	3.58,s	52.0
13		60.7	28	1.22,d,7.0	9.5

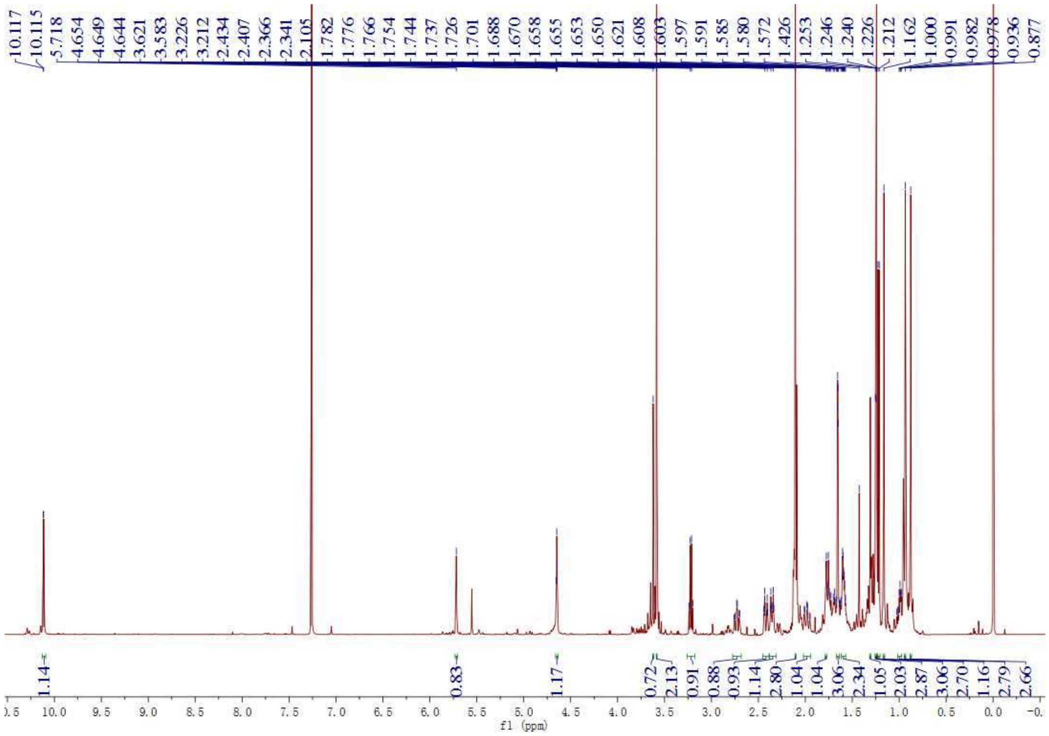

Figure S20. ^{1}H NMR spectrum of andrastin A (**4, in keto form**) in $CDCl_3$

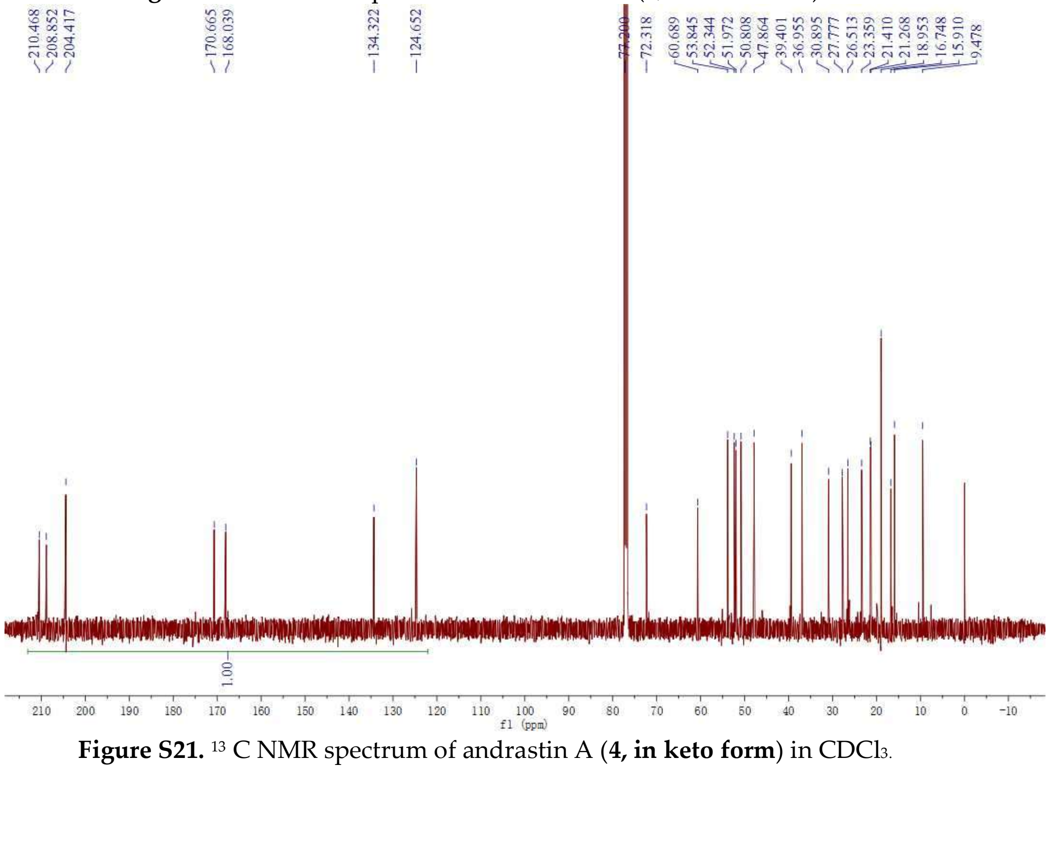

Figure S21. ^{13}C NMR spectrum of andrastin A (**4, in keto form**) in $CDCl_3$.

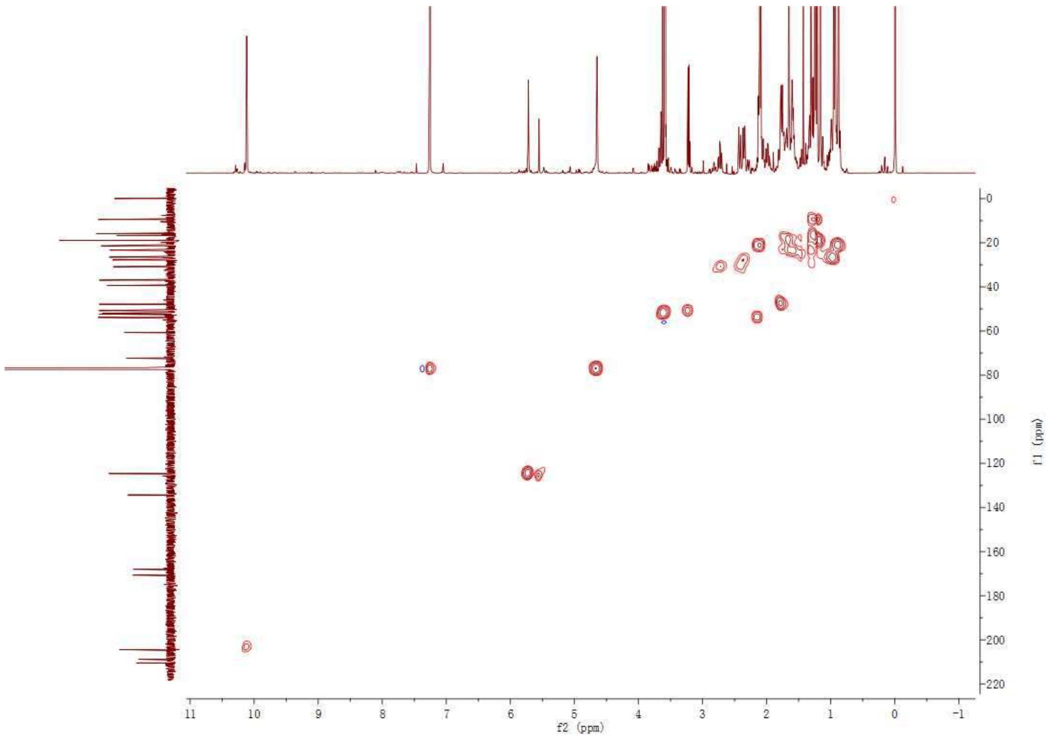

Figure S22. HSQC spectrum of andrastin A (**4, in keto form**) in $CDCl_3$.

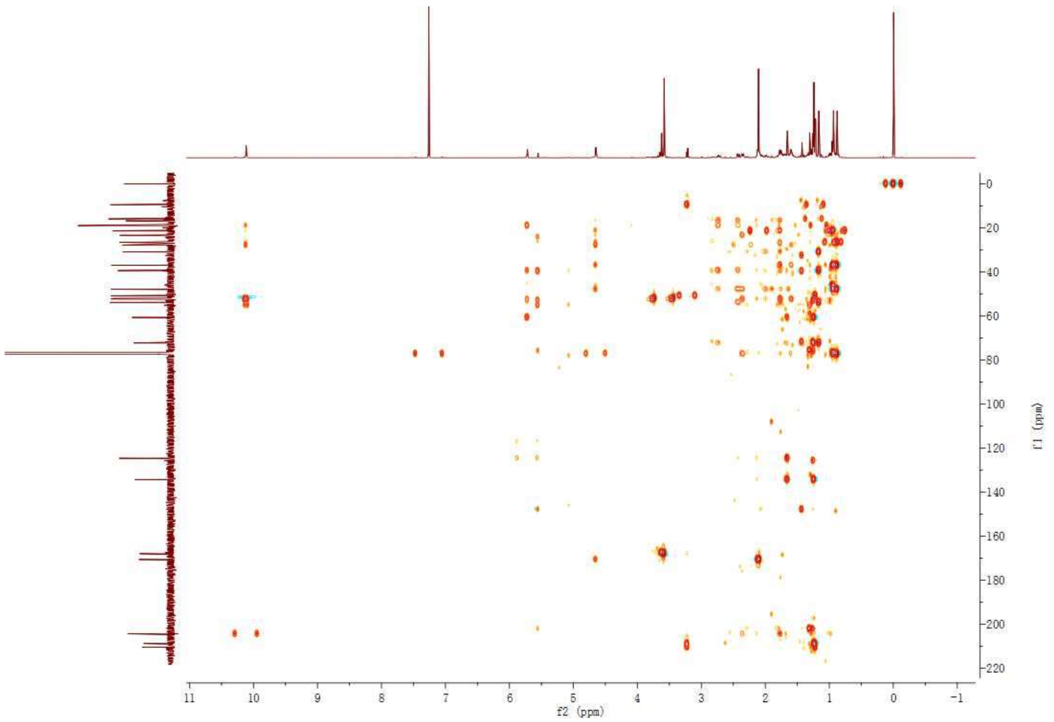

Figure S23. HMBC spectrum of andrastin A (**4, in keto form**) in $CDCl_3$.

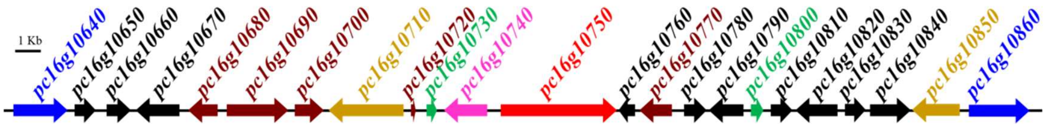

Figure S24. Proposed biosynthetic gene cluster (*pc16g10640-pc16g10860*) of compounds **1–3**

Table S4. Putative ORFs and predicted functions of gene cluster *pc16g10640-pc16g10860.*

Penicillium chrysogenum (Wisconsin 54-1255)	Predicted function
pc16g10640	Fungal specific transcription factor
pc16g10650	NAD(P)-dependent dehydrogenase
pc16g10660	Dyp-type peroxidase
pc16g10670	Oxidoreductase/Monooxygenase
pc16g10680	Hypothetical protein
pc16g10690	Hypothetical protein
pc16g10700	Hypothetical protein
pc16g10710	Major facilitator superfamily
pc16g10720	Hypothetical protein
pc16g10730	Decarboxylase
pc16g10740	O-methyltransferase
pc16g10750	Nonreducing PKS
pc16g10760	Dehydratase
pc16g10770	Hypothetical protein
pc16g10780	Reductase/Dehydrogenase
pc16g10790	Oxidase
pc16g10800	Decarboxylase
pc16g10810	Peroxisomal short-chain alcohol dehydrogenase
pc16g10820	Oxidoreductase/Monooxygenase
pc16g10830	Short-chain alcohol dehydrogenase
pc16g10840	Salicylate 1-monooxygenase
pc16g10850	Major facilitator superfamily
pc16g10860	Fungal specific transcription factor

Table S5. Antitumor activities (in vitro IC_{50}, μM) of compounds **1–3.**

**Compounds**	**IC_{50} (μM)**				
	U87 MG	NCI-H1650	HT29	A498	HL-60
**1**	22.6	42.2	41.8	28.5	37.2
**2**	>50.0	>50.0	30.8	>50.0	16.2
**3**	47.0	>50.0	43.2	>50.0	22.7
**Secalonic acid D**	5.64	4.93	1.46	8.88	0.41
